# Pesticides and Health in Vegetable Production in Kenya

**DOI:** 10.1155/2015/241516

**Published:** 2015-12-10

**Authors:** Ibrahim Macharia

**Affiliations:** Department of Agribusiness Management and Trade, Kenyatta University, P.O. Box 43844–00100, Nairobi, Kenya

## Abstract

This paper investigates the determinants of pesticide-related cost of illness (COI) and acute symptoms, using a balanced panel of 363 farmers interviewed from seven major vegetable producing districts of Kenya. Finding shows that the incidences of pesticide-related health impairments have increased. Variation in number of symptoms and symptom severity significantly explained COI. The personal protective equipment (PPE), education level, record keeping, and geographical location considerably determined health impairments. Encouraging the proper use of PPE and record keeping of pesticide use could greatly reduce poisoning cases and COI.

## 1. Introduction

The health effects of pesticide use have become one of the major public health problems worldwide. In developing countries, frequent exposure to pesticides by farmers and farm workers is very common [1–3]. The frequent exposures to pesticides result in both short-term (acute) and long-term (chronic) illnesses. Scientifically confirmed pesticide-related acute illnesses include headaches, stomach pains, vomiting, skin rashes, respiratory problems, eye irritations, sneezing, seizures, and coma [4]. The chronic illnesses include cancer, asthma, dermatitis, endocrine disruption, reproductive dysfunctions, immunotoxicity, neurobehavioral disorders, and birth defects [5–13]. Furthermore, deaths resulting from direct exposure to pesticides are also common [14].

The World Health Organization (WHO) and the United Nations Environment Program (UNEP) estimate pesticide poisoning rates at 2-3 per minute [14]. The largest numbers of pesticide poisonings and deaths are said to occur in developing countries [15]. It has been argued that pesticide-related health issues constitute a serious threat to development and can easily reverse or undermine the gains made in agricultural growth [16]. Poor access to health services and the inability of medical professionals to recognize pesticide-related morbidity raise further concerns [17]. Although pesticide-related poisoning is still not as high or more pronounced in Africa as in Asia, it is a growing problem as the increasing intensification of agricultural production with more widespread use of pesticides will result in possible increase in pesticide poisoning [18].

In Kenya, pesticide use and farmers health have been documented by some empirical studies [19–21]. However, these studies were based on a snap shot of cross-sectional surveys and a clear trend of poisoning is not well understood. In addition, only two studies looked at the determinants of pesticide-related acute poisoning symptoms among farmers [19, 20]. However, the problem is that pesticide poisoning effects on human are not random but rather depend on other unobserved characteristics such as genetic characteristic. Such effects cannot be captured with cross-sectional data as utilized in the above studies. Thus the true underlying causal relations may be very different, either larger or smaller, compared with those noted in those researches.

The objective of this paper therefore is to examine the incidences and the determinants of acute pesticide poisoning among vegetable farmers in Kenya controlling for unobserved heterogeneity.

## 2. Methods

### 2.1. Surveys and Data

**Figure 1:**
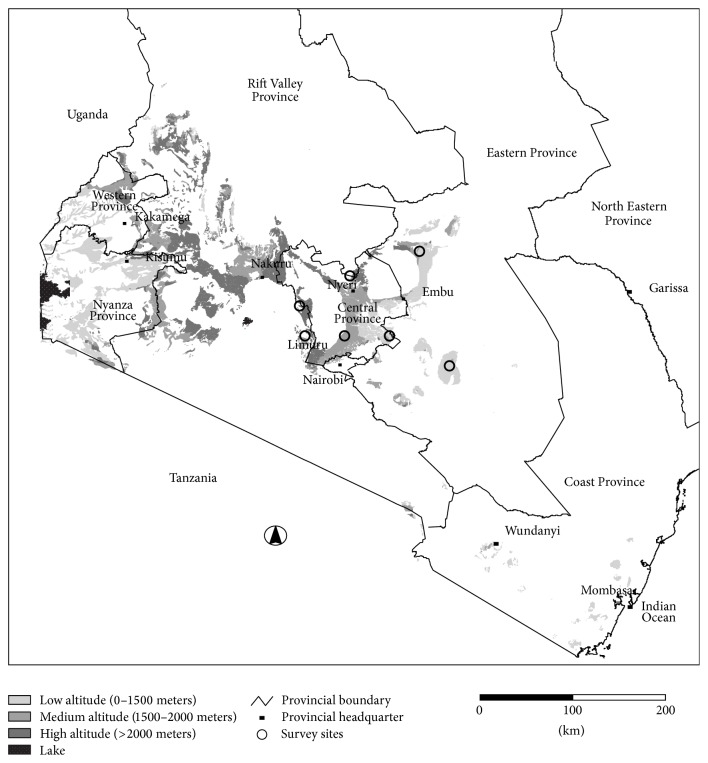
Study sites. Source: this study based on GIS mapping of potential vegetable production areas.

The study was conducted in seven major vegetable producing districts of Central Province (Kiambu, Kirinyaga, Murang'a, Nyandarua, and Nyeri North) and Eastern Province (Makueni and Meru Central) of Kenya (Figure 1) in the year 2005 with follow-up visits in 2008.

The 2005 survey comprised 839 interviews from the Diamondback moth biological control impact assessment survey (“DBM” with 295 farmers) and the Global Good Agricultural Practices (“GLOBALGAP” with 544 farmers) assessment survey. GLOBALGAP (formerly known as EUREPGAP) is a private sector body that sets voluntary standards for the certification of agricultural products around the globe. In both surveys, a multistage sampling procedure was used to select districts, sublocations, and farmers, respectively. First, districts were purposely sampled according to intensity of vegetable production and agroecological zones. Lists of farmers that were compiled by extension workers at sublocation level served as sampling frame from which 839 farmers were randomly sampled by probability proportional to size (PPS) procedure.

Sampled farmers were then monitored in one cropping season and were trained in record keeping of their production activities by trained enumerators. The trained enumerators under direct supervision of the researcher visited the farmers to check the records and transferred the information to the survey questionnaire.

Due to budget constraints, the 2008 survey was a recall survey of a random subsample of 425 farmers among the 839 farmers. However, we only obtain 363 balanced data set after the 2 years of study.  Table 1 displays the distribution of farmers in the sampled districts.

The semistructured questionnaires employed covered a wide range of topics, such as cropping systems, demographics, common farming practices, pesticide use and handling practices, and type and quantities of pesticides sprayed. Health symptoms investigated were specified as those that only began during the spraying operation or within 24 hours after spraying. Additional information collected included the following: number of times the symptom occurred, workdays lost partially or completely due to the health impairment, medication taken by victims, and direct costs due to the symptoms, that is, pharmacy cost, consulting fees, and indirect costs such as travel expenses to and from health centre and dietary expenses resulting from illness like drinking milk or taking honey.

### 2.2. Analytical Framework

As discussed earlier panel data setup was used to control for the unobserved heterogeneity. In general, panel data model offers some distinct advantages over the cross-sectional data analysis. Greene [22] concluded that the main advantage of panel data is that one can formally model the heterogeneity across groups that are typically present in panel data. Baltagi [23] confirms this in his statement that the first benefit of panel data is “controlling for individual heterogeneity.” Additional benefits of using panel data are the ability to capture both cross section and time-series variation in the dependent variable and measure not only the effects that observable variables have on the dependent variable, but also the effects of relevant unobservable or nonmeasurable influences.

A general panel regression model is presented as

(1) yit=α0+βXit+γZi+Vit,Vit=εi+μit,

where *y*_*it*_ is the response of the dependent variable (in our case this is the cost of illness (COI) or number of acute symptoms) for the *i*th farmer in the sample at the *t*th year. *α*_0_ is an intercept that may be different for each point in time, and *β* and *γ* are vectors of coefficients. *X*_*it*_ is the set of *K*-vector of time-variant covariates for the *i*th farmer at the *t*th year and *Z*_*i*_ is another set of predictor variables that do not vary over time (time-invariant), for example, gender and location. *V*_*it*_ is the error term, which is decomposed into *ε*_*i*_ and *u*_*it*_. *ε*_*i*_ is regarded as the combined effect on* y* of all unobserved variables that are constant over time (time constant unobserved heterogeneity such as cognitive ability and motivation), and *μ*_*it*_ represents the idiosyncratic error term (what is unaccounted for in the model) and varies over individual farmers and over time.

The two main methods of dealing with *ε*_*i*_ are to make the random effects (RE) or fixed effects (FE) assumption. Random effects assumed that *ε*_*i*_ are random variables that is, *ε*_*i*_ is i.i.d. (0, *σ*_*ε*_^2^) and that Cov(*x*_*it*_, *ε*_*i*_) = 0, while with fixed effects, *ε*_*i*_ are assumed to be potentially correlated with *X*_*it*_. In fixed effect regressions we cannot estimate the effects of time constant covariates as these are normally cancelled out by the within transformation. Thus, classic fixed effects approaches do not produce any estimates of the effects of variables that do not change over time. Moreover, in some cases fixed effects estimates may have substantially larger standard errors than random effects estimates, leading to higher *p* values and wider confidence intervals. In addition, fixed effects estimates use only within-individual differences, essentially discarding any information about differences between individuals unlike random effects that used information both within and between individuals. Thus, if predictor variables vary greatly across individuals but have little variation over time for each individual, then fixed effects estimates will be rather imprecise. When neither the cross-sectional unit nor times have significant effects, all of the data can be pooled and one can have the constant coefficients model.

The analysis was implemented in two steps. First, the COI model was estimated to evaluate the determinants of health costs among the vegetable farmers. Cost of illness was computed as the sum of farmer-reported medical treatment costs to clinics and private physicians, the opportunity cost of workdays lost to illness, travel costs to and from health facility, time spent in traveling, and the cost of home-based health care. In the second stage, the principal factors associated with the pesticide poisoning symptoms were examined seeking ones that are relevant at policy recommendation.

### 2.3. The Models 

#### 2.3.1. Cost of Illness Model

In previous studies, the health costs of pesticides were modeled using a Logarithmic regression model [19]. In this study the estimation of the determinants of health costs was modeled using a censored random effects Tobit model (Xttobit), since zero costs from respondents who had suffered pesticide-related illnesses but incurred no costs were considered. Using a Logarithmic model would have required adding a small unity value as log of zero is undefined. Estimation of dependent variables results in biased estimators in linear models [24]. The structural equation in the Tobit model is represented as

(2) $$\begin{array}{c} y_{it}^{*}=x_{it}\beta +\varepsilon_{i}+u_{it}, \end{array}$$

where *ε*_*i*_ ~ *N*(0, *σ*_*μ*_^2^) and *y* ^*∗*^ is a latent variable that is observed for values greater than *T* and censored otherwise. The observed* y* is defined by the following measurement equation:

(3) $$\begin{array}{c} y_{it}=\left\{\begin{array}{cc} y^{*} & \text{if }y^{*}>T\\ T_{y} & \text{if }y^{*}\leq T. \end{array}\right. \end{array}$$

In the typical Tobit model, we assume that *T* = 0; that is, the data are censored at 0.

For the empirical model, the explanatory factors for the model explaining health costs incorporate four broad classes of variables, namely, those related to health (number of acute symptoms and symptoms severity), farmer characteristics variables (age, education, and gender), farm management variables (farm size (proxy for wealth), GLOBALGAP certification, and record keeping), and location control (district dummies) (see (4)). Variable definitions and descriptive statistics are presented in Table 2.

It is hypothesized that the number of acute symptoms, symptom severity, age, and farm size are positively associated with the health costs, while a negative association is expected for level of education, GLOBALGAP certification, and record keeping. The direction of the effect of gender on health costs is not clear a priori.

It is anticipated that young farmers may have a higher tendency to protect against pesticides exposure and consequently reduce the pesticide-related acute symptoms and associated health costs. Increased education is also expected to reduce health costs because farmers are more likely to read pesticide labels and follow the recommendation, again reducing the exposure and the acute symptoms. Likewise, GLOBALGAP certification and record keeping can result in a more judicious use of pesticide and higher tendency to protect against pesticide intoxication resulting in reduced acute symptoms:

(4) HEALTHCOST=fTACUTE,SEVERE,AGE,AGESQ,EDUCATION,GENDER,FARMSIZE,GLOBALGAP,RECORD,District Dummies, YEAR 2008 Dummy).

*Acute Symptoms Model*. The determinants of the number of acute symptoms were modeled as random effects. A Negative Binomial Regression model (Xtnbreg) was chosen to account for overdispersion, since the equidispersion assumption that has to be met with the Poisson model was violated; that is, the variance was larger than the mean and just over two-thirds of the counts were zero. When there is overdispersion, the Poisson regression is not appropriate because the standard errors estimated are biased downward and the *p* values are small and spurious [25].

A Negative Binomial Regression model is a count data model and a good facet of the model is that the Poisson model is nested within it [25]. However, the assumption of the standard Poisson model that the variance of the dependent variable is equal to the mean is not binding for the negative binomial model [26]. Negative Binomial Regression model deals with the problem of overdispersion by assuming that *y*_*it*_ has a negative binomial distribution, which can be regarded as a generalization of the Poisson distribution with an additional parameter allowing the variance to exceed the mean. The negative binomial function can be presented as

(5) fyit ∣ μit,εi=Γμit+yitΓμitΓyit+1εi1+εiyitεi1+εiεi,

where Γ is the gamma function, parameter *u*_*it*_ is assumed as both the mean and the variance, and parameter *ε*_*i*_ is assumed constant over time for each individual, while the mean and variance of *y*_*it*_ are given by

(6) Eyit=εiμit,var⁡yit=1+εiεiμit.

Under this model, the ratio of the variance to the mean is 1 + *ε*_*i*_ that can vary across individuals but is constant over time. The basic idea for this model is that the predictor information is related to the rate of the response to increase or decrease in counts.

For the empirical model, the acute symptom model aggregates skin irritation, diarrhea, sneezing, headache, dizziness, vomiting, stomach poisoning, blurred vision, eye irritation, and backache episodes incurred by the farmer during and/or soon after spraying pesticide as the dependent variable. For the explanatory variables, the medical literature indicates that the type and severity of pesticide poisoning depend on the toxicity of the pesticides, amount of pesticides involved in the exposure, and route of exposure [27]. The model accounted for these factors. In addition, in order to understand farm management variables that can affect pesticide poisoning, GLOBALGAP certification and record keeping were included. Furthermore, following Antle and Pingali [4], Wilson and Tisdell [28], and Asfaw [19], other control variables under farmer characteristics, that is, age, gender, education, and geographical location, were also included (7).

A priori, it is anticipated that WHO class Ia, Ib, and II pesticides are positively correlated with incidences of acute poisoning whereas negative correlation can be expected with category III and U pesticide. Pesticides in WHO Ia, WHO Ib, and WHO II are very harmful, while WHO III and WHO U are less harmful [29].

Age could increase acute symptoms, as older farmers may be less concerned about health effects of pesticides. As already mentioned in cost of illness model it is expected that pesticide-related acute symptoms decrease with the increase in level of education, GLOBALGAP certification, record keeping of production activities, and appropriate use of personal protective equipment:

(7) TACUTE=fAGE,AGESQ,EDUCATION,GENDER,GLOBALGAP,RECORD,NPEST,PWHOIab,PWHOII,PWHOIII,PWHOU,COAT,GLOVE,GUMBOOT,MASK,District Dummies,YEAR 2008 Dummy.

The models were estimated using the random effect estimator as the Hausman test showed the fixed effects were not correlated with the regressors. All variables were cross-checked for the problem of multicollinearity, through the simple correlation matrix and variance inflation factor (VIF). The highest correlation coefficient was 0.32 and VIF were by far less than three, indicating that correlation between explaining variables could not affect the estimation of coefficients. Likewise, for endogeneity none of the independent variables was suspected to be explained within the equation in which they appeared. Misspecifications of the models were also checked using a regression specification error test [30]. In respect to the robustness of the Negative Binomial Regression model, a Poisson model was first fitted and the likelihood ratio test together with the statistical evidence of overdispersion indicated that the Negative Binomial Regression model was preferred to the Poisson model. In addition, to check the robustness of all the models other restricted models were estimated in which subsequently insignificant variables were dropped. The statistical quality of the models and the direction of the signs did not change, and the coefficients deviated only marginally.

## 3. Empirical Results and Discussion

### 3.1. Descriptive Statistics of Variables Used in Empirical Estimations

Table 2 summarizes the main descriptive statistics comparing 2005 and 2008 with *t*- and *z*-tests for the main variables investigated. The results showed that the incidences of pesticide-related acute illness had increased by over 70%. By cross-check although not indicated in the table the analysis showed that only 45% of the farmers consequently reported the effect once more in 2008 showing a high rate of new episodes cases. However, the number of symptoms per farmer dropped by almost half in 2008. In terms of frequency of symptom occurrence, headache and sneezing were reported as the main symptoms in both surveys. Dizziness as one of the major neurological effects of pesticide exposure was also found to have doubled in 2008. These symptoms have been associated with pesticides acute poisoning [27]. They are also consistent with other studies of pesticides exposure on farmers' health elsewhere [31–33].

A total of 62 pesticides products, comprising 36 active ingredients formulated singly or in mixture, were used to control various vegetable pests in 2005. The number increased slightly to 66 products in 2008 with 44 active ingredients in the formulations. However, close analysis showed that 19 new products were applied in 2008, implying that 15 products of those used in 2005 were dropped. The commonly used products included dimethoate (WHO II), used by 48% of farmers, lambda cyhalothrin (WHO II, 27%), cymoxanil (WHO II, 22%), cypermethrin (WHO II, 22%), cyfluthrin (WHO Ib, 20%), mancozeb (WHO U, 18%), and deltamethrin (WHO II, 14%).

For minor poisoning, many farmers used home remedies such as milk, lemon juices, honey, and herbs. The medicines from the local pharmacy shops which were sometimes painkillers were bought in cases where the symptoms of illness were mild and farmers visited the health clinic if the symptoms either persisted or became serious; that is, the victim was unable to talk, walk, or see or vomited continuously. This evidence seems to suggest that many farmers treat acute pesticide effects as minor problems that do not warrant medical attention. Although in only about a quarter of the poisoning cases a physician was consulted, this cost component accounts for the largest share of the total cost of treatment.

The health cost almost doubled in 2008 as compared to 2005. On average, health cost was estimated at US$ 6.55/farmer/season for 28% of the farmers who reported pesticide-related illnesses. These costs equal 47% of mean household pesticides expenditures in 2008. Considering all the farmers this translates to a mean of US$ 1.77/farmer/season and assuming two crop seasons per year the costs amount to US$ 3.54/farmer/year. However, the true health costs are likely to be much higher because the costs arising from chronic diseases resulting from long-term pesticides exposure were not considered, as this would have required more detailed medical assessments. Moreover, only costs directly involving family members were reported; costs occurring to hired farm laborers were not included. Furthermore, other “costs” to restore health status completely and nonmonetary costs like suffering and income lost by family members assisting in seeking treatment were not captured [34, 35]. In addition, preventive costs associated with precautions taken to reduce exposure such as wearing protective equipment were not considered because they were mainly improvised from old clothing or pieces of cloth wrapped around the nose and mouth to reduce inhalation exposure. The cloths were also used for other purposes like spraying on coffee and other farm work and it was difficult to specify those used for spraying pesticides on vegetable crops alone. However, the combined mean of personal protective equipment used increased by 43%, with the largest increment noted for gumboots. Over 20% of farmers also paid wage premiums of up to 32% above the normal wage to hired labor for spraying pesticides, which were normally paid in cash.

Comparison with other studies conducted in developing countries shows similar results. Pingali et al. [36] showed that 58%–99% of the farmers exposed to pesticides had at least one health effect symptom in Indonesia, Philippines, and Vietnam. In Tanzania farmer spending on health due to pesticide and exposure ranges between US$ 0.018 and 116 in a year [37]. In West Africa, the economic value of pesticide-related health costs equals US$ 3.92/household/season in the case of cotton-rice systems [38]. Zimbabwe cotton growers incur a mean of US$ 4.73 in Sanyati and US$ 8.31 in Chipinge on pesticide-related direct and indirect acute health effects [2]. In Sri Lanka, cost to farmers from pesticide exposure equals 10 weeks' income [39], while in India the average annual welfare loss to an applicator from pesticide exposure amounts to US$ 36 [40]. The immediate costs of a typical intoxication (medical attention, medicines, and days of recuperation) equaled the value of 11 days of lost wages in Ecuador [41].

Pesticide application rate/hectare/season also increased by 47%. Comparison between the years for the specific farmers who participated in the DBM survey showed that many farmers had reduced the pesticides application rate by 8%, while the GLOBALGAP surveyed farmer had increased by 40%. Similar findings in support of the reduction of pesticide use were reported by Jankowski [42] and Löhr et al. [43] where farmers in the study areas with DBM biocontrol (*Diadegma semiclausum*) reduced pesticide applications with others even stopping spraying altogether. The increase in application rate by GLOBALGAP farmers can partially be explained by the low number of farmers who were certified at the time of survey and the failure of the farmers certified in 2005, to maintain their certification status; that is, certified farmers dropped from 18% to 7%, with only 31% of the farmers maintaining their certification for 2008.

### 3.2. Model Estimations

#### 3.2.1. Cost of Illness Estimation

The estimation results of the Tobit models with the health costs as dependent variable are reported in Table 3. Result shows that health costs are positively associated with number of symptoms and symptoms severity, which implies that an increase in any of these variables spontaneously influences positively the health costs, holding other factors constant.

The finding that the GLOBALGAP certification tends to decrease the health cost could indicate that the certified farmers use adequate safety precautions or use low toxic pesticides, which generally reduce the health impairments and thus decrease costs. It could also be that these farmers are able to use the minimum treatment possibilities.

Among the farmers' characteristics variables, that is, age, education, and gender, none had any discernible effect on health costs. In addition, farm size though considered as an indicator of wealth does not have a direct effect on health costs, though it has the correct sign. Perhaps it could be because farms do not present “liquid cash” that can be accessed immediately in time of need. In addition, no direct association was found between record keeping and the health costs.

District controls are insignificant, so location does not directly affect the health costs. When the model was reestimated (restricted) by dropping insignificant variables, the estimates of the coefficients were robust.

#### 3.2.2. Acute Symptoms Estimation

Given the critical contribution of pesticide-related acute symptoms to the health costs as indicated in Table 2, the principal determinants of these symptoms are reported in Table 4.

The model shows that pesticide-related acute symptoms increase significantly with the number of pesticide products handled. This is not surprising, given that different pesticide products require different application rates and have different levels of toxicity. In addition, handling different pesticide products can increase incidences of symptoms since an interaction between pesticides can lead to unknown toxic chemical reactions [44]. Likewise, although the coefficients for pesticides in WHO Iab and WHO II are insignificant, they are positively correlated with acute symptoms whereas negative correlation is observed with WHO III and WHO U pesticides. The significant negative sign of the variable “record keeping” suggests that the probability of pesticide-related illnesses is less for farmers who keep records. In general, record keeping of pesticide products handled, their application dosage, application techniques, and production activities enabled farmers to be more judicious on pesticides use and higher tendency to protect them. With records, a farmer can also see how well she/he is managing production operations and can identify the strengths and weaknesses in those activities.

The level of education reduces the probability of reported symptoms, which implies that farmers with a higher education level are more knowledgeable and therefore have a better understanding of the dangers posed by pesticides. In previous studies, however, the contrary effect was found because respondents with higher knowledge were more likely to report more health symptoms [2].

The use of personal protective equipment particularly the use of a coat/apron and facemask significantly reduced the number of symptoms. Exposure to pesticides is often attributed to a failure to use protective equipment [34]. The positive sign of the use of boots although insignificant seems perverse and alarming at first glance. However, as the researcher had observed in the field, the improper use, that is, putting the trouser inside the boots, may offer a partial explanation of this apparently perverse result. This finding is analogous to that found by Ohayo-Mitoko et al. [45], where use of gumboots was associated with high* acetyl cholinesterase* inhibition.* Acetyl cholinesterase* is enzyme that breaks down acetylcholine (ACh) into choline and acetic acid. It is released onto the sarcolemma of muscle fibers and destroys ACh after the ACh has combined with receptors on the muscle fiber. Thus, it prevents continued muscle contraction in the absence of additional nervous stimulation.

Location control for agroecology and differences in institutional settings shows that farmers in the districts of Kiambu, Meru Central, Makueni, Nyandarua, and Nyeri North experience significantly high cases of pesticide ascribed health symptoms as compared to Kirinyaga (base). Perhaps this is due to the use of protective equipment by farmers located in Kirinyaga.

Contrary to the expectations, the analysis does not support the hypothesis of a significant influence of GLOBALGAP certification on the outcome of health, but the variable has the correct signs. Once again, the low number of farmers who were certified and the failure of the certified farmers to maintain their certification may be the cause of the insignificance. The hypothesis that gender and age have a stronger relation to the acute symptoms is also not supported by the results.

The likelihood ratio test used to assess the statistical quality of the model showed that the model was statistically valid, that is, dispersion parameter alpha was greater than zero. The reduced model with only the variables that had a significant effect on the dependent variable shows that the statistical quality of the model does not differ much and the directions of the coefficient are identical, suggesting the robustness of the model.

## 4. Conclusions and Recommendations

The results of the study give indications of increase of pesticide-related health impairments with over 70% new episodes. The most frequently reported symptoms were sneezing, dizziness, headache and blurred vision, and skin irritations. The result further shows that farmer loses on average US$ 3.54/farmer/year on pesticide-related indirect health costs. These costs are significantly explained by variation in number of symptoms and severity of the symptoms. Pesticide-related acute symptoms increase significantly with the number of pesticide products handled and considerably reduce with level of education, use of PPE, and record keeping. These findings hint at some important points for policies aiming to reduce pesticide poisoning among vegetable farmers. Firstly, the results support the already widely known notion that proper use of PPE (coat/apron and facemask) reduces the pesticide-related impairment. Encouraging of PPE and record keeping of pesticide use activities by farmer is thus recommended.

Future efforts to measure pesticide-related health costs should cover the health costs of all individuals exposed to pesticides, for example, entire public, consumers, and hired workers, and also incorporate pesticide-induced chronic illnesses and deaths.

## Figures and Tables

**Table 1:** Regional distribution of survey respondents.

Province	District	Main vegetable crops		Previous surveys (2005)	Number of farmers sampled (2008)	Balanced data (farmers)
		Domestic	Export			
Central	Kiambu	Cabbages, kales, and spinach		48	27	19
	Kirinyaga	Peas, tomatoes	French beans	155	74	66
	Muranga	Tomatoes, kales	French beans	51	24	21
	Nyandarua	Cabbages, potatoes		119	52	49
	Nyeri North	Peas, cabbage, onions, and carrots	French beans	277	116	107

Eastern	Makueni	Cabbages, kales	Asian vegetables^a^	49	22	8
	Meru Central	Peas, tomatoes, cabbage, and onions	French beans	140	110	93

^a^Brinjals, karella, dudhi, okra, turia, valore, and aubergine.

Source: this study.

**Table 2:** Descriptive statistics of variables used in empirical estimations (*N* = 726).

Variables	Definition	Unit	Mean^a^		*t* or *z* stat.^b^
			2005	2008	
Dependent variables					
TACUTE^c^	Number of symptoms	Count	1.89 (0.13)	1.09 (0.03)	−7.07^*∗∗∗*^
TACUTE	Number of symptoms	Count	0.38 (0.48)	0.37 (0.03)	−0.15
HEALTHCOST^c^	Cost of illness	US$	4.15 (1.70)	7.98 (1.57)	1.57
HEALTHCOST	Cost of illness	US$	0.84 (0.35)	2.72 (0.58)	2.80^*∗∗*^

Farmer characteristics variables					
AGE	Age of the farmer	Years	43.19 (0.66)	46.18 (0.67)	6.30^*∗∗∗*^
AGESQ	Age of the farmer (years squared)	Years	2024.43 (62.80)	2292.64 (66.85)	65.21^*∗∗∗*^
EDUCATION	0 = none; 1 = preprimary; 2 = primary; 3 = secondary; 4 = college	Ordinal	2.45 (0.05)	2.51 (0.04)	1.09
GENDER	Male	1/0	0.70 (0.02)	0.70 (0.02)	0.00
EXPERIENCE	Farming experience	Years	18.42 (0.74)	20.56 (0.07)	2.38^*∗∗*^

Health-related and pesticide exposure variables					
HEALTH	Farmer reported a symptom	1/0	0.20 (0.02)	0.34 (0.02)	4.26^*∗∗∗*^
SEVERE	1 = mild; 2 = severe; 3 = very severe	Ordinal	1.11 (0.08)	1.59 (0.36)	1.22
PWHOIab	WHO Ia and WHO Ib (extremely hazardous)	g	8.79 (2.32)	33.55 (12.79)	1.92^*∗∗*^
PWHOII	WHO category II (moderately hazardous)	g	129.87 (10.15)	432.63 (25.20)	10.97^*∗∗∗*^
PWHOIII	WHO category III (slightly hazardous)	g	18.95 (3.39)	166.12 (19.23)	7.45^*∗∗∗*^
PWHOU	WHO category U (no hazard)	g	79.87 (7.47)	167.79 (16.86)	4.87^*∗∗∗*^
PESTHA	Total amount applied	g/ha/season	1,473.00 (201.82)	2,124.87 (118.28)	2.97^*∗∗∗*^
NPEST	Pesticide products	Count	2.89 (0.09)	3.32 (0.08)	3.37^*∗∗∗*^
COAT	Wear coat/apron	1/0	0.49 (0.03)	0.71 (0.02)	6.06^*∗∗∗*^
GLOVE	Wear gloves	1/0	0.26 (0.02)	0.35 (0.02)	2.49^*∗∗*^
GUMBOOT	Wear boots	1/0	0.26 (0.02)	0.89 (0.02)	17.35^*∗∗∗*^
MASK	Wear facemask	1/0	0.24 (0.02)	0.40 (0.02)	4.36^*∗∗∗*^
TPPE	Protective equipment	Count	2.81 (0.07)	4.00 (0.11)	10.85^*∗∗∗*^

Farm management variables					
FARMSIZE	Total farm size	ha	1.46 (0.08)	1.06 (0.05)	−4.46^*∗∗∗*^
GLOBAL-GAP	GLOBALGAP certified farmers	1/0	0.07 (0.01)	0.19 (0.02)	0.15
RECORD	Records keeping	1/0	0.71 (0.02)	0.32 (0.01)	−10.47^*∗∗∗*^

All monetary variables for example health cost were adjusted (normalized) to US$ of 2008 to take account of inflation. US$ = 72 KSh (2005) and 75 KSh (2008).

^a^Figures in parenthesis are standard errors.

^b^Statistical significance at the 0.01 (*∗∗∗*), 0.05 (*∗∗*), and 0.1 (*∗*) levels of probability. Categorical variables were analyzed using *z*-test.

^c^With only farmer who reported the health impairment.

Source: this study.

**Table 3:** Tobit model for cost of illness estimations.

Model	Unrestricted		Restricted	
Variables	(Coefficient)^a^	*z*-value	(Coefficient)^a^	*z*-value

TACUTE	7.45 (4.08)^*∗∗*^	1.83	6.20 (2.00)^*∗∗∗*^	3.10
SEVERE	9.01 (2.52)^*∗∗∗*^	3.58	11.07 (2.17)^*∗∗∗*^	5.12

AGE	−0.48 (1.09)	−0.44		
AGESQ	0.01 (0.01)	0.61		
EDUCATION	1.46 (2.36)	0.62		
GENDER	−2.84 (4.33)	−0.66		
FARMSIZE	3.25 (2.88)	1.13		

GLOBALGAP	−21.75 (3.40)^*∗*^	−1.62	−18.71 (7.47)^*∗∗*^	−2.50
RECORD	−1.08 (4.89)	−0.22		

KIAMBU	2.50 (10.31)	0.24		
MAKUENI	−15.08 (17.25)	−0.87		
MERU CENTRAL	1.65 (8.71)	0.19		
MURANGA	−5.48 (11.75)	−0.47		
NYANDARUA	−5.61 (8.81)	−0.64		
NYERI NORTH	6.62 (8.47)	0.78		
YEAR 2008	7.93 (9.15)	0.87		

Constant	−23.23 (29.41)	−0.79	−19.54 (4.45)^*∗∗∗*^	−4.39
Log likelihood	−464.10		−549.55	
Wald *χ* ^2^/LR *χ* ^2^	40.18^*∗∗∗*^		43.22^*∗∗∗*^	

^a^Figures in parenthesis are robust standard errors, statistically significant at the 0.01 (*∗∗∗*), 0.05 (*∗∗*), and 0.1 (*∗*) levels of probability.

Source: this study.

**Table 4:** Binomial Regression model for the acute symptoms estimations.

Model	Unrestricted		Restricted	
Variables	(Coefficient)^a^	*z*-value	(Coefficient)^a^	*z*-value

AGE	0.04 (0.04)	1.13		
AGESQ	−0.00 (0.00)	−1.21		
EDUCATION	−0.16 (0.07)^*∗∗*^	−2.13	−0.14 (0.07)^*∗*^	−1.94
GENDER	−0.10 (0.16)	−0.67		
GLOBALGAP	−0.33 (0.29)	−1.11		

RECORD	−0.44 (0.17)^*∗∗∗*^	−2.57	−0.55 (0.15)^*∗∗∗*^	−3.77

NPEST	0.09 (0.05)^*∗∗*^	1.88	0.10 (0.05)^*∗∗*^	2.39
PWHOIab	0.00 (0.00)	1.28		
PWHOII	0.00 (0.00)	0.68		
PWHOIII	−0.00 (0.00)	−0.28		
PWHOU	−0.00 (0.00)	−0.13		

COAT	−0.29 (0.16)^*∗*^	−1.82	−0.29 (0.15)^*∗∗*^	−2.03
GLOVE	−0.26 (0.21)	−1.23		
GUMBOOT	0.32 (0.23)	1.36		
MASK	−0.35 (0.20)^*∗*^	−1.74	−0.39 (0.17)^*∗∗*^	−2.30

KIAMBU	1.69 (0.36)^*∗∗∗*^	4.67	1.63 (0.32)^*∗∗∗*^	5.20
MAKUENI	1.74 (0.49)^*∗∗∗*^	3.55	1.50 (0.46)^*∗∗∗*^	3.35
MERU CENTRAL	1.18 (0.31)^*∗∗∗*^	3.82	0.95 (0.25)^*∗∗∗*^	3.77
MURANGA	0.64 (0.46)	1.40		
NYANDARUA	0.90 (0.34)^*∗∗∗*^	2.66	0.80 (0.28)^*∗∗∗*^	2.81
NYERI NORTH	0.93 (0.30)^*∗∗∗*^	3.07	0.79 (0.24)^*∗∗∗*^	3.26
YEAR 2008	−0.05 (0.21)	−0.23		
Constant	−1.22 (1.06)	−1.15	−0.01 (0.48)	−0.02
Log likelihood	−518.85		−535.52	
Wald *χ* ^2^	73.74^*∗∗∗*^		60.96^*∗∗∗*^	

^a^Figures in parenthesis are robust standard errors, statistically significant at the 0.01 (*∗∗∗*), 0.05 (*∗∗*), and 0.1 (*∗*) levels of probability.

Source: this study.
